# Antiradical Activity of Beetroot (*Beta vulgaris* L.) Betalains

**DOI:** 10.3390/molecules26092439

**Published:** 2021-04-22

**Authors:** Maciej Spiegel, Andrzej Gamian, Zbigniew Sroka

**Affiliations:** 1 Department of Pharmacognosy and Herbal Medicines, Wroclaw Medical University, Borowska 211A, 50-556 Wroclaw, Poland; zbigniew.sroka@umed.wroc.pl; 2 Ludwik Hirszfeld Institute of Immunology and Experimental Therapy, Polish Academy of Sciences, Rudolfa Weigla 12, 53-114 Wroclaw, Poland; andrzej.gamian@hirszfeld.pl

**Keywords:** antioxidants, density functional theory, betalains, reactive oxygen species, beetroot, phytochemicals, *beta vulgaris*, superfood, amaranthaceae, antiradical activity

## Abstract

Flavonoids, phenolic acids, and anthocyanidins are widely studied polyphenolics owing to their antiradical activity. Recently, beetroot dyes have drawn an attention as possible radical scavengers, but scant information can be found on this topic. In this study selected compounds were investigated using computational chemistry methods. Implicit water at physiological pH was chosen as the environment of interest. Betalains’ dissociation process and electronic structure were examined, as well as the reactivity in six pathways against some common radicals, such as hydroxyl, hydroperoxide, superoxide, and nitric oxide. The study showed that all carboxyl groups are dissociated in the given conditions. The dissociation process impacts the electronic structure, which has consequences for the overall activity. Highly stabilized conjugated structures favor the electron–accepting type of scavenging reactions, primarily by a radical adduct formation mechanism. Betanidin and indicaxanthin were found to be the most promising of the compounds studied. Nevertheless, the study established the role of betalains as powerful antiradical dietary agents.

## 1. Introduction

Oxidation of biological structures is commonly documented as a factor in the incidence of serious age–related morbidities [1]. Reactive oxygen species (ROS) are the key initiators of these processes, but sulphur [2] and nitrogen reactive species [3] have also recently attracted researchers. The bulk of ROS are radicals whose prooxidant activity originates from the high amount of energy they carry due to the unpaired electron on the valence shell. Such electronic configuration is very unstable, forcing a radical to stabilize itself. This can be achieved either by detaching a hydrogen atom, by accepting an electron from the attacked molecule, or by forming an adduct. The capacity to eradicate oxidants is the primary role of biological compounds present in the cell matrix, e.g., glutathione [4]. When their concentration decreases, biological structures become the subject of an undesirable oxidation process and oxidation stress occurs.

Beetroot (*Beta vulgaris* L.) is one of the oldest and most widely cultivated, along with potatoes, plants in the rural areas. It is remarkably important for poor communities, where beetroots tend to be a common dietary element because they are inexpensive and easy to sustain. Recently it has also gained an attention as a ‘superfood’: a natural food product rich in nutrients. Its composition is reflected in a variety of beetroot activities: antioxidant, anticancer, and antimicrobial [5]. An abundance of both micro- and macroelements can be found inside them, as well as polyphenols [6], vitamins [7], and betalains [8]. Betalains belong to the colorful, water-soluble, nitrogen pigments found commonly in the *Amaranthaceae* family [9]. Some of them are used as food coloring additives, e.g., betanin as an E162. Betalains are mainly stored in the root, but they can be present in the other parts of the vegetable too. Based on the structure and color, betacyanins (indole structured; red) and betaxanthins (aliphatic-amine structured; yellow) can be distinguished (Table 1). While the pigmentation type is similar to that presented by anthocyanins, biological synthesis of betalains differs as it goes through tyrosine and L–DOPA semiproducts [10]. Experimental assays have shown that betalains demonstrate good antiradical and antioxidant activity, either alone or as a part of an extract [11,12,13,14].

Nowadays, at least four elementary and three multi–step mechanisms of antiradical activity can be identified [15]. Each of them is characterized by specific indices that are perceived as estimators of the relative antioxidant reactivity. As a simple example, when comparing two radical scavengers, the one which exhibits a lower value of the studied index is considered as the better one. Electronic structure methods, particularly density functional theory (DFT), are frequently used in such analysis, allowing one to inspect the chemistry of radicals and the antiradical activity of phytochemical compounds [16,17,18].

The preferred mechanism is the one for which the radical generated is less reactive than initial reactants and the energy surplus is stabilized. The question of which one is favored depends on the solvent, the radical concentration, and the chemical structure of the antioxidant. Polar and protic solvents are typically suitable milieu for electron-related channels due to the first-step product, an ion. It is stabilized by a charge separation and, as a result, less energy is required to initialize the reaction. On the other hand, lipid phases do not support them; instead, hydrogen ones, such as hydrogen atom transfer or radical adduct formation, are chosen [19,20]. Any of these activities arise from the presence of hydroxyl groups bonded by conjugated bonds. This helps the antioxidant radical product to lower its energy due to the resonance. [16] For example, the scavenging of nitric oxide by caffeic acid has been shown to benefit primarily from the sequential electron transfer mechanism [21], whereas gallic acid tends to flush out hydroxyl radicals mostly as a part of the sequential proton loss–hydrogen atom transfer path [17]. Most of the betalains do not have OH groups, with certain exceptions, e.g., betalain or indicaxanthin. Instead, these compounds are rich in electron density, which indicates that the electron-related mechanisms should be responsible for the exhibited activity [22].

Owing to the above information, the present study was carried out to fill a hole and lay the groundwork for future research. Within it, we used DFT methods to establish thermodynamic properties of antiradical activity expressed by some common beetroot betalains in order to investigate it at the molecular level. The study focused on several common reactive species, including hydroperoxyl (OOH^•^) and hydroxyl (OH^•^) radicals, nitric oxide (NO^•^), and superoxide ion ($O_{2}^{•-}$), which are particularly important mediators of oxidative stress. Furthemore, since the chemical structure of betalains lets us find them in at least three distinct states depending on the pH of the solvent, estimating the form preferred in the studied system is critical. The experimental values of betalains’ p*K*_a_ were scarcely accessible at the time of writing the paper, being either inaccessible or restricted to a single deprotonation step. Thus, prior to the main course of the study, it was determined which forms have the highest molar fraction at pH = 7.4—computational methods are able to predict p*K*_a_ values with a very limited error [23,24]. The significance of protonation state stems from the experimental data that have already confirmed a strong impact of deprotonation on antiradical activity, especially for this group of compounds. [11,25,26,27]. Additionally, knowing how the molar distribution changes with pH can aid in adjusting it in order to obtain a desired form of betalain in experimental assays or more practical circumstances such as formulations of beetroot-derived food additives (like new antioxidants). In the end, using the Donator–Acceptor Map [28], an activity comparison with vitamins and anthocyanins was performed, providing some practical view into the antioxidative power of betalains.

## 2. Results

### 2.1. Dissociation Path and p*K*_a_ Estimation

Deprotonation routes (Table 2) have been calculated according to the methodology provided in Materials and Methods. The Gibbs free energies for each step are presented in Supplementary Materials (Table S1).

Aside from indicaxanthin, it was found that the first two deprotonations were connected with carboxyl groups bonded to the tetrahydropyridine ring. The most acidic one is bound to the C6 atom and demonstrates p*K*_a_ values not greater than 1.80. The second most acidic carboxyl is related to C2, with p*K*_a_ approximately 3.5 for all compounds but betacyanins.

**Figure 1:**
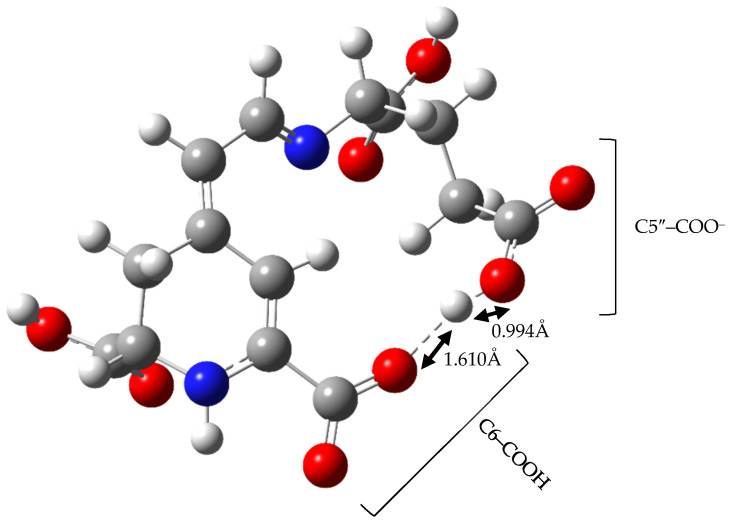
Geometry of Vulgaxanthin II C5″ Ion.

Third and further deprotonation routes identify first the remaining carboxyl groups and then hydroxyl groups. Interestingly, we found it impossible to estimate the value of Gibbs free energy for deprotonation of vulgaxanthin II’s C5″–carboxyl group due to hydrogen atom movement during the optimization process. In the resultant structure of an ion, the distances between hydrogen, negatively charged oxygen of carboxylate, and oxygen from carboxyl connected with C6 were found to be 0.994 Å and 1.610 Å respectively (Figure 1). Freezing the O–H bond of the C6 moiety resulted in the imaginary frequency whose eigenvector suggested hydrogen movement to negatively charged oxygen of the C5″ residue. For this reason, it has been assumed that the dissociation of C6 is energetically favored to the degree that the work required for the hydrogen atom swap between two carboxyls can be overcome.

The exception to the proposed deprotonation routes is indicaxanthin where the dissociation pattern is C2″→C6→C2. While the gap of the first deprotonation p*K*_a_ between C2″ and C6 is very small (0.07) and may be only an error, C2″ still remains more acidic than C2.

After all, the variations in p*K*_a_ values between successive deprotonation stages are not greater than 2 units, unless the hydroxyl hydrogen is detached, which is the case for betanidin or betaxanthin. There, it differs in at least 5 units. It can also be noted that the more carboxyl groups are present, the more acidic is the betalain. For each subsequent dissociation, the p*K*_a_ values of the remaining ones rise.

**Figure 2:**
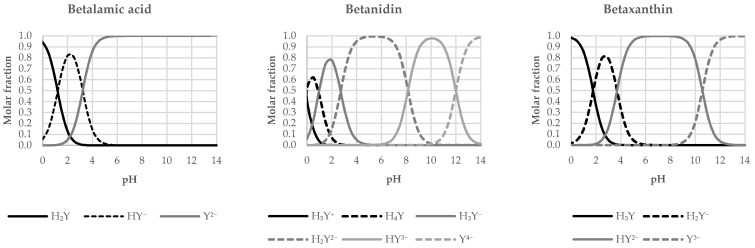

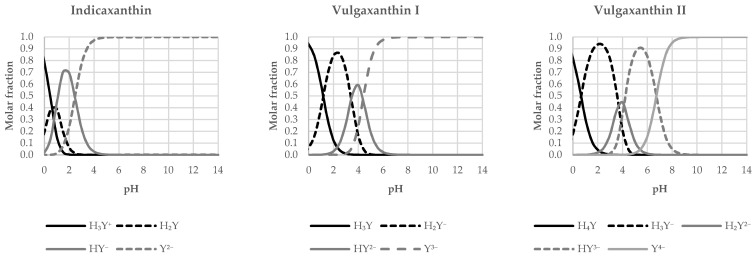
Distribution Diagrams for the Studied Betalains.

Based on the data presented, the distribution diagrams were plotted (Figure 2) at 0–14 pH intervals. It can be observed that most of the investigated compounds have fully dissociated carboxyl groups prior to passing the pH value 7.4 the exception is vulgaxanthin II (p*K*_a4_ = 6.72) and therefore a margin of HY^3−^ form (~17.4%) could still be present at physiological pH. Similarly, betanidin (p*K*_a4_ = 8.14) is present as a mixture of H_2_Y^2−^ and HY^3–^ at a ratio of 8:2. For those not fully dissociated, the last deprotonations are associated with the hydroxyl residues. Starting from Section 2.2, any time the name of betalain is used its most favored form at physiological pH shall be considered unless otherwise specified.

### 2.2. Electronic Structure Analysis

Basic properties of the electronic structure have been investigated. These are vertical ionization potential (vIP), vertical electron affinity (vEA), and HOMO–LUMO gap (HLG), presented in the first three numerical columns of Table 3. Using Koopman’s theorem, vIP is defined as a negative of the highest occupied molecular orbital (HOMO) energy while EA is a negative of the lowest unoccupied molecular orbital (LUMO) energy. The antioxidant’s low ionization potential (high HOMO energy) and the radical’s high electron affinity greatly facilitate electron transfer onto the latter one, increasing the propensity of the scavenging reaction. On the other hand, if an antioxidant has higher electron affinity (high LUMO energy), it may become an electron acceptor.

While the ionization potentials are within comparatively limited values (average 5.25 eV), the electron affinities are noticeably lower and vary to a degree. Because of that it can be stated that HLG depend only on vEA values, which are additionally inversely proportional to the energy gap between these orbitals. Betanidin has vEA of 3.17 eV, whereas for betaxanthin or vulgaxanthins it is approximately 2.0 eV. This means the difference is in approximately 23.0 kcal/mol (reference value of 1 eV). Betaxanthin (3.25 eV) and vulgaxanthins (both ~3.15 eV) can be clustered as representatives of the compounds with the largest HLG, while betalamic acid (2.82 eV), indicaxanthin (2.47 eV), and betanidin (2.09 eV) can be clustered as representatives of the lowest one.

On the basis of vIPs and vEAs, the electrodonating (ω^–^) and electroaccepting (ω^+^) powers were calculated to evaluate charge–exchange properties. Electrodonating power is negatively correlated with the donating potential, while electroaccepting power is positively correlated with the acceptance capacity. Again, betanidin, indicaxanthin, and betalamic acid can be clustered as the compounds with the highest electrodonating and electroaccepting power values. This means that they are less likely to donate charge and more susceptible to accepting it. The remaining betalains show almost identical values of these indices and, in the same manner as the vIP–vEA relationship, the values of ω^–^ are higher than those for ω^+^.

### 2.3. Reactivity Indices

The enthalpies of all antioxidant activity indices were determined, as well as Gibbs free energies for reactions with the chosen radicals. Full data can be found in Supplementary Materials. Enthalpies of water-solvated species—electrons and protons—were taken from the paper of Marković et al [29]. Thermochemical parameters of the solvated hydrogen atom were calculated directly with unrestricted ωB97–XD/aug–cc–pVQZ [30].

#### 2.3.1. Radical Adduct Formation (RAF)

The antioxidant is capable of undergoing radical reactions without losing any electrons or hydrogen, but by forming a more stable adduct. This mechanism is known as radical adduct formation. It was found to be determined by the antioxidant structural features and was observed among compounds rich in delocalized multiple bonds, such as carotenoids with π–electron density distributed across the molecule [31]. The radical type is also important in this mechanism: those that are small and electrophilic, such as OH^•^ [32,33,34], have the highest tendency for RAF.

The condensed Fukui functions [35] for radical attack were assessed in order to establish the desired binding site of a radical (Table S2). According to the findings, the adducts were constructed and Gibbs free energies calculated for each of them. The obtained valued were then used to calculate RAF energetics (Table 4). As can be seen, RAF convey negative values for hydroxyl radical reaction centers unless the tetrahydropyridine nitrogen atom is examined. Hydroxyl radicals willingly bind to the selected carbon atoms, with energetics of up to –34.0 kcal/mol. Apart from these, only bonding with hydroperoxide is desired, and only for the first carbon atom of the side chain. Thermochemical studies for superoxide ion and nitric oxide RAFs reveal noticeably positive values, and thus their feasibility is doubtful. The exceptions are $O_{2}^{•-}$ for C1′ of betaxanthin, indicaxanthin, and both vulgaxanthins, for which ΔG < 10 kcal/mol. Under these circumstances other products of superoxide scavenging via RAF should also be considered.

The formation of a new bond with tetrahydropyridine nitrogen is not desirable at all even though estimated Fukui functions achieved high values for this atom. Our goal was to check whether electrons of betalains are delocalized to a degree capable of counteracting electron density deformation on nitrogen orbitals due to the adduct formation. Unfortunately, it does not seem to be favored in the scope of thermochemistry. The question arises as to whether reactions can be driven by kinetics, especially for the hydroxyl radical. Additionally, for some sites it was impossible to optimize the product (imaginary frequencies were present) even when unpruned grids had been used.

#### 2.3.2. Hydrogen Atom Transfer (HAT)/Proton Coupled Electron Transfer (PCET)

The presented mechanism is a one-step reaction that involves antioxidant hydroxyl hydrogen abstraction by the radical during a homolytic breakage in the hydroxyl O–H bond. As a result, the separated hydrogen forms a new bond with the radical, neutralizing it. Bond dissociation enthalpy (BDE) is a well-established descriptor used to assess the system’s relative feasibility in the HAT mechanism. It was found to be more preferred in nonpolar solvent or nonionic aqueous solutions, whereas PCET predominates in reactions involving highly electronegative reactants [36]. Another difference is that, in HAT, the hydrogen atom is transferred as a single entity, while in PCET, the proton and the electron shift simultaneously but independently, and thus may come from different orbitals. According to the research, electron density interactions in HAT are a form of three-electron delocalization along the reaction path, while PCET includes four-electron delocalization [37].

Among the studied betalains, only betanidin and betaxanthin have hydroxyl groups and can scavenge the radicals via this channel. For the first compound, the bond dissociation enthalpies vary by ~5.5 kcal/mol and C5″–O–H cleavage seems to be simpler (Table 5). It is just marginally higher for betaxanthin than betanidin. Overall, the BDE span is in a narrow range from 72.3 kcal/mol to 79.9 kcal/mol. Concerning reactivity towards radicals, one can see that neutralizing hydroxyl and hydroperoxide radicals is thermochemically affordable, whereas superoxide and nitric oxide are still a challenge.

#### 2.3.3. Single Electron Transfer (SET)

An electron is either transferred to or removed from the reactive species during single electron transfer, resulting in its neutralization and stabilization. Therefore, the best determinants of activity are adiabatic ionization potential (aIP) and adiabatic electron affinity (aEA). This simple reaction is involved in more complicated mechanisms including sequential proton loss–electron transfer or sequential electron transfer–proton transfer, but it is often though to occur on its own for certain antioxidants [21,38]. In this article, special emphasis is placed on SET since most betalains cannot scavenge radicals through hydrogen–driven mechanisms, in consequence limiting their activity in lipophilic solvents where ion products are not favored.

At first sight, it can be noted that Gibbs free energies of electron transfer (ET, Table 6), understood as adiabatic ionization potentials or adiabatic electron affinities, with respect to produced antioxidant, are positive for electron-donation (upper) and negative for electron-acceptance steps (lower). The anion radical is readily produced by every antioxidant studied here. Analyzing reactions with the radicals, only the conversion from ${\mathrm{OH}}^{•}$ to ${\mathrm{OH}}^{-}$ yields negative value, and thus is thermodynamically feasible. The hydroxyl radical is the most electrophilic species in our set, which explains the observed energetics of its reduction process. Scavenging of $O_{2}^{•-}$, which contrary to hydroxyl radical is nucleophilic, follows the ET pattern, so the Gibbs free energies for electron donation pathways are lower. Energy changes of reactions with nitric oxide are associated with a change in the free energy of at least 64.0 kcal/mol for the cation radical product (vulgaxanthin II) and in 78.3 kcal/mol for the anion radical product (betanidin). Overall, the explanation for the observed tendency is rather clear: withdrawing an electron from the radicals would result in formation of ${\mathrm{OH}}^{+}$, ${\mathrm{OOH}}^{+}$, or ${\mathrm{NO}}^{+}$. Such species are much more unstable than the radicals they derive from, which consequently is expressed in the observed ΔG values.

#### 2.3.4. Sequential Electron Transfer–Proton Transfer (SET–PT)

The SET–PT mechanism consists of two simple steps: first, SET happens, and an electron transfers from the antioxidant to the radical, and then the proton is passed between them, resulting in an antioxidant radical. As with SET, an adiabatic ionization potential is assessed to determine the propensity of the reaction first step. For the second stage of the process, the proton dissociation enthalpy (PDE) is calculated.

From Table 7 it can be seen that the first step is a strongly limiting one, with an ionization potential of more than 80 kcal/mol greater than that of succeeding proton dissociation enthalpies. As electron transfer enthalpies have already been presented in the previous subsection, they will be omitted here. We introduced the abbreviation PT (proton transfer) to distinguish the energetics of reactions with radicals from the antioxidants’ intrinsic properties. The proton transfer step is strongly exergonic for all of the post–ET reactions. Thus, if a sufficient amount of the energy required to initialize the first step is given, the reaction will be successfully brought to an end.

#### 2.3.5. Sequential Proton Loss Electron Transfer (SPLET)

SPLET, which can be thought of as a reverse-ordered SET–PT, is another typical antiradical channel. According to the recent studies, SPLET is a representative mechanism of the bulk phytochemical antioxidants. [16,18,39]. It consists of two steps: first, a deprotonation (represented by a proton affinity, PA), and second, an electron transfer to a radical. It is apparent that the basicity of the medium increases the reaction ratio greatly, while the acidity moves the equilibrium down to an undissociated form of antioxidant, lowering the mechanism viability.

The enthalpies of the proton affinity (Table 8) can be ordered as follows: betanidin–C5″ (32.9 kcal/mol) < betanidin–C6″ (36.7 kcal/mol) < betaxanthin–C4″ (40.3 kcal/mol). Comparing the values obtained for the second step with ET presented earlier, one can see that electron transfer from an ion requires less energy than from a neutral molecule. Moreover, the computed aIP values are very close to each other regardless of the hydron abstraction site. Looking at the Gibbs free energies of SPLET second step, all of the radicals exhibit positive values, much greater for nitric oxide and superoxide. As a consequence, no thermodynamically favored reactions can be indicated. However, PA is sufficiently small that, given certain conditions, hydroxyl and hydroperoxide neutralization will be possible.

#### 2.3.6. Sequential Proton Loss Hydrogen Atom Transfer (SPLHAT)

The last mechanism, sequential proton loss–hydrogen atom transfer (SPLHAT), was initially proposed as a major pathway of anthocyanidin cation scavenging activity towards DPPH. [40] It behaves similarly to SPLET, except that instead of an electron, the hydrogen atom is transferred in the second step. That is why, for the first and second step, it tends to be represented by proton affinity and proton dissociation enthalpy, respectively. Clearly, this specification insists that two hydrogens are available and, as a result, in our case is limited just to betanidin.

The outcomes are presented in Table 9. We will skip a discussion on proton affinity since it has already been addressed in the SPLET mechanism subsection. BDEs of proceeding hydrogen transfers are associated with higher enthalpies, inversely proportional to PA values. Estimated Gibbs free energies of the second step suggest that the hydrogen radical is easily neutralized through SPLHAT. While the scavenging of superoxide radical is not anticipated, hydroperoxide and nitrogen oxide could be flushed away in this manner.

## 3. Discussion

As we mentioned at the beginning of this paper, details on the dissociation of betalains are sparse. About 50 years ago, Piatelli et al. [41] measured the p*K*_a_ of indicaxanthin with potentiometric titration and estimated the value at 3.3; for comparison, the lowest one derived from our calculations is 2.45. Next, we found that betanin, an aglycone of betanidin, had both experimental p*K*_a_ values of C2 and C6 approximately ~3.4, with C2″ within the range of 1.5–2.0 [42]. In the theoretical research on the same compound, Tutone et al. [43] determined p*K*_a_ of tetrahydropyridine carboxyl groups using three different methods. The best results were 2.9 for C6 (Jaguar/DFT) and 2.96 (Epik)/3.12 (Marvin) for C2. Our computational findings for betanidin yield different values—0.10 and 0.95 respectively, and 2.71 for the pyrrole carboxyl group—although the potential effect of glycosylation on p*K*_a_ should not be ignored. Further attention must be paid to the topic of betalain dissociation constants through conducting laboratory assays that could affirm or refute the theoretical dissociation routes. Nevertheless, this does not influence the main course of our study since all of the chosen betalains are dissociated prior to reaching pH = 7.4.

Betalains’ acidity is not surprising and can be easily clarified if delocalization is taken into account. Upon the dissociation, the resultant carboxylate is firmly stabilized by conjugated double bonds that extend from the semi-unsaturated ring to the side chain and, in the case of betanidin or indicaxanthin, up to the side ring. Second order perturbation theory analysis given by the NBO framework [44] yields findings supporting significant interactions of type π→π*, n→π* and hyperconjugation n→σ*. They are found within every betalain backbone structure and, using betalamic acid for example, can be labeled as follows:

- the delocalization in conjugated bonds system: πC6–C5→π*C4–C1′ (25.30 kcal/mol), πC6–C5→π*C_carboxyl_–O1_carboxyl_ (16.00 kcal/mol), πC4–C1′→π*C6–C5 (13.33 kcal/mol); :O1_carboxyl_ →σ*C6–C_carboxyl_ (20.48 kcal/mol);
- nitrogen lone pair delocalization: :N1→π*C6–C5 (44.26 kcal/mol);
- C6–carboxyl resonance: :O1_carboxyl_→σ*C_carboxyl_–O2_carboxyl_ (34.79 kcal/mol), :O2_carboxyl_ → π* C_carboxyl_– O1_carboxyl_ (53.85 kcal/mol)

Their effect on deprotonation is easily noticeable in the betanidin example in which the delocalization extends to the side chain and in which p*K*_a1_ is –0.10 (for contrast, p*K*_a_ of HNO3 is roughly –1.30).

Some aspects require extra consideration from a biochemical point of view. First of all, the cationic and neutral structures disappear quickly after they reach the pH of gastric acid. This renders them totally irrelevant to biological systems. Next, the antiradical activity of betanidin and betaxanthin will decrease with increasing basicity as the hydroxyl groups’ dissociation leaves these compounds with electron-related mechanisms as the only way of activity. Finally, it is remarkably important to bear in mind that even small fractions of certain species can exhibit detectable activity, particularly when radicals are scavenged by mechanisms that are not accessible to other forms of that antioxidant (e.g., scavenging of hydroperoxide by HAT or SET–PT).

Apart from the effect on dissociation, the electronic structure influences the overall reactivity of betalains. As we noted at the outset, the absence of hydroxyl hydrogens does not actually imply that the compounds are inactive. The strong antiradical potency found during experimental studies confirms the role of electron-related pathways in betalain activity. The ABTS^•+^ assay of betalamic acid conducted by Gandia–Herrero et al. [11] yielded a TEAC value of 2.7 and betanidin was observed [27] to have a value over 6. These values are higher than for most of phenolic acids [45], flavonoids [46], and carotenoids [47], and because they were measured at pH = 7, they indicate that betalains are also potent antioxidants through their electron–related channels. Since betalamic acid is our “lowest” reactive molecules and betanidin is our “highest” reactive molecule, we may infer that other betalains may have similarly high activity, yet that should be examined further in experimental studies.

To establish this computationally we inspected the electronic structure of the ion that is most abundant at physiological pH. The vertical ionization potential results obtained for our set of compounds are almost identical and can be understood as they oxidize themselves equally. The obtained vertical electron affinity implies that the ability to accept electrons varies among them and would be the key determinant of the activity. The most useful inference to be reached is that both vIP and vEA have a positive meaning that betalains will behave in a binary manner: by obtaining and giving electrons, but with different efficiency. This is controlled by orbital interactions with radicals’ singly occupied molecular orbital (SOMO) and the distance between betalain’s HOMO and LUMO. The energy gap between the antioxidant frontier orbitals and the radical’s SOMO is meaningful since the reaction is known to operate even more smoothly when this value is small, and the interacting orbitals appear closer together on the energy diagrams.

**Figure 3:**
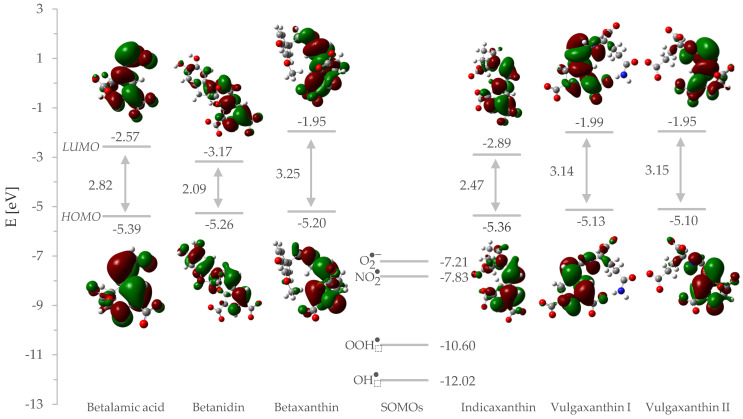
Energy Diagram for HOMOs and LUMOs of the Studied Betalains and SOMOs of the Radicals Along with Their Graphical Representation.

Betanidin, indicaxanthin, and betalamic acid have the highest EA values of the compounds studied, at the same time displaying the smallest HLG values (Figure 3). Large EA means that the electronic system is capable of accepting additional electron density, while small HLG allows π–electrons to be distributed to higher energy levels, thereby lowering the energy of the structure and making excited states more accessible.

Since the energy difference between HOMO and LUMO is inversely proportional to the aromaticity, betalains can be sorted by delocalization as follows: betanidin > indicaxanthin > betalamic acid > vulgaxanthin I = vulgaxanthin II > betaxanthin. This is in line with the second order perturbation theory analysis carried out earlier, confirming that indole (betanidin) and pyrrole (indicaxanthin) as well as the electron-withdrawing aldehyde group (betalamic acid) activates the structure for electron acceptance better than the aliphatic amine backbone of the remaining compounds. This theoretical outcome was evidenced in the absorbance spectroscopy study [27] in which betanidin displayed a maximal excitation wavelength (542 nm) among 15 betalain derivatives studied. Utilizing frontier molecular orbital theory, the SOMO energies are below all HOMO energies, indicating electrophilic attack of radicals on the orbital with the largest electron density.

**Figure 4:**
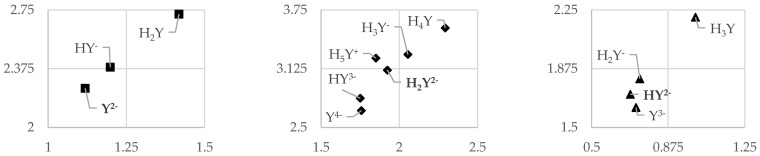

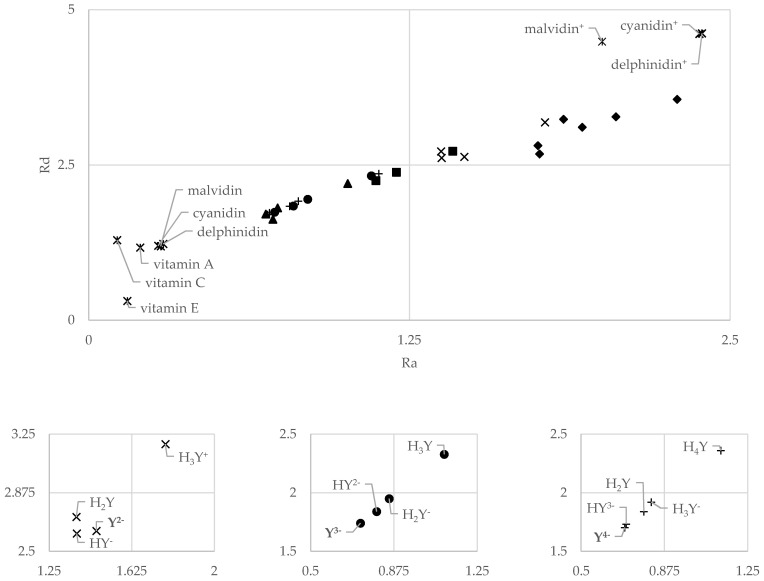
Donator–Acceptor Maps for Betalains. The Numbers in Bold Typeface Indicate the Form Favored at pH = 7.4.

The electron donation (Rd) and acceptance indices (Ra) were calculated, and the Donator–Acceptor Maps (DAM) were plotted (Figure 4). Reference to anthocyanins and vitamins has been considered [48]. Betanidin (◆), the only representative of betacyanins, can easily be distinguished as a powerful radical scavenger, being located in the upper-right quarter (good antireductant), nearby cationic anthocyanidins. On the other hand, betalamic acid (■) and betaxanthins appear to change their activity to stronger electron donors (good antioxidants). Conversion from the upper-right to the lower-left quarter occurs at each corresponding point of dissociation and is linearly correlated with almost all of the compounds, except indicaxanthin (**✕**) and betanidin. No major variations were found for vulgaxanthin I (●), vulgaxanthin II (**+**), or betaxanthin (▲).

All of the compounds analyzed fall between the vitamins or neutral anthocyanidins and cationic forms of the latter. Since León-Carmona [23] has found that anthocyanidin cations are discarded just after pH = 4, we should contend that our set of betalains represents the best antireductant activity from the plotted set of compounds. Those in the upper-right quarter have been shown to have the highest degree of bond conjugation. Like Martinez et al. [49], we can see that strong delocalization increases the antireductant capacity of betalains but reduces the antioxidative one. On the other hand, along with the consecutive dissociation, this profile shifts and betalains are more likely to donate the electrons than accept them. Yet, this role is still poorer than that of vitamins or neutral anthocyanidins.

**Figure 5:**
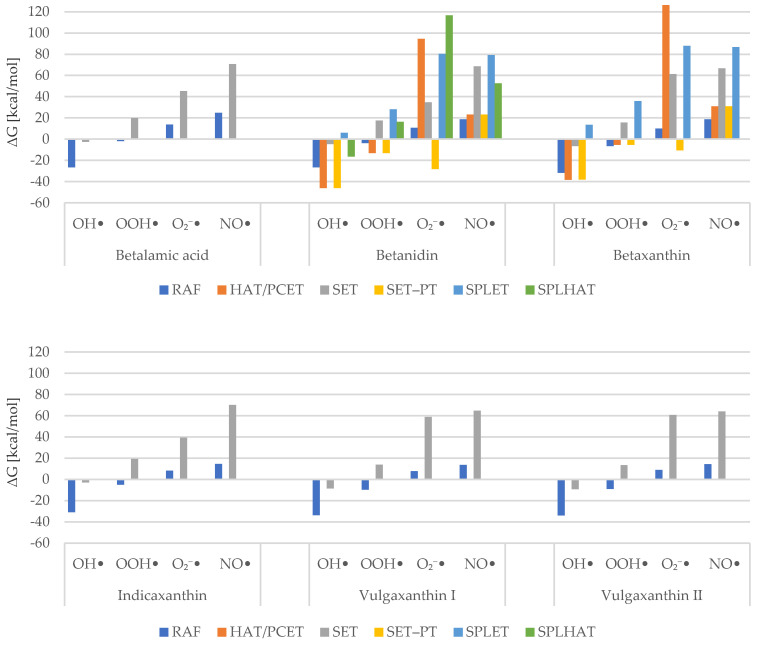
Sum of Gibbs Free Energies for Examined Mechanisms.

Our step-by-step calculations for the possible mechanism of action allow us to map the energies as a chart seen above as Figure 5. It presents the sums of Gibbs free energies for each mechanism of antiradical activity against a specific radical. The lowest values were taken as compound representatives. We compared the results with other phytochemical radical scavengers of known activity.

It can be inferred that the hydroxyl radical is, in fact, the most readily scavenged ROS. All HAT/PCET, SET, and RAF channels are exergonic in at least –38.0 kcal/mol for the first two and in at least –26.0 kcal/mol for the third mechanism. Gibbs free energy of the radical adduct formation is considerably smaller than that found for gallic acid [17]. HAT/PCET and SET–PT routes, available for betanidin and betaxanthin, are also lower in energetics than those found for phenylacetate derivatives [50]. Interestingly, it was found that SPLET is associated with positive Gibbs free energy. This is somewhat surprising, as it is commonly claimed to be a desirable mechanism for phytochemicals in polar solvent [39,50,51,52].

Hydroperoxide radical scavenging appears to be spontaneous for RAF, HAT/PCET, and SET–PT channels. The first was found to occur more willingly for vulgaxanthins, while the other compounds displayed a comparatively limited intensity of this mechanism. Betanidin seems to scavenge OOH^•^ at a higher yet similar pace, for both HAT/PCET and SET–PT paths. In contrast, these pathways have around the same values as RAF for betaxanthin, which may make them insignificant. Amić et al. [53] determined Gibbs free energies for double HAT and double SPLET mechanisms of several polyphenols. Comparing these with our findings indicates that betaxanthin scavenges hydroperoxide using the HAT channel at an energy level close to that demonstrated by homoprotocatechuic acid, catechol, quercetin, and caffeic acid. It is also smaller than that exhibited by phenylacetate derivatives [50]. Overall, our data suggest that neutralizing this radical is mainly favored through RAF. Gallic and sinapic acids have not been found to be capable of doing so, at least in the thermodynamic scope. [17,54] Our HAT/PCET results are similar to the findings of Marino et al. [17] for gallic acid. Sinapic acid [54], which appears to scavenge hydroperoxide more effectively than betalains, is another reference. Moreover, in the first place, SPLET reactions are exergonic, whereas here this mechanism is associated with positive Gibbs free energy, and in the second place, SET–PT is not spontaneous, whereas here it seems to be.

In the course of our research, it was found that superoxide radical and nitric oxide are the most resistant ROS. Superoxide ions tend to be favorably scavenged via SET–PT, but RAF values are small enough to make that mechanism considerable too. We also obtained high values of HAT and significantly negative values of SET–PT, whereas for hydroxyphenylacetates they were found to be much lower and much larger, respectively. [50] Finally, none of the Gibbs free energies was negative for nitric oxide. The lowest values are displayed once again by the RAF mechanism. However, HAT/PCET might be involved in its neutralization.

**Figure 6:**
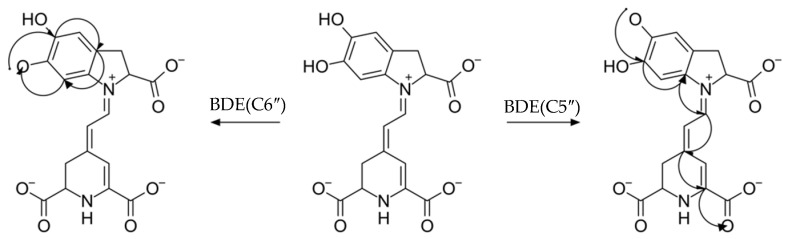
Visualization of Electron Flow After Bond Dissociation in Betanidin.

Interestingly, for hydrogen-based pathways such as HAT/PCET, SPLET, and SPLHAT, we found that the reactivity is dependent on hydrogen atom accessibility, similarly to the one from our previous study. [16] Namely, studying flavones and flavonols, we found a situation where two adjacent C3′ and C4′ hydroxyl groups manifested notable variations in BDE. Such a situation happened for, e.g., luteolin. We concluded that the reason lies in two factors: the delocalization and hydrogen bonding. For the C4′ radical product, the electron density was able to reach the chromone ring, while C3′ was confined to the B–ring only. The same is true for betanidin (Figure 6): a product formed from C5″ after hydrogen is detached is quickly delocalized all the way down to the tetrahydropyridine ring, reaching the carboxylate. On the other hand, C6″ has no such capability. Finally, not only delocalization but also the catechol moiety facilities hydrogen-related reactions due to the hydrogen bond between hydroxyl and the formed product, which thus decreases the system energy. [55] Since betaxanthin does not have such opportunities, BDE and PA values are slightly higher.

Our study confirms the previously stated hypotheses that betalains’ activity originates from electron resonance and donating an electron is less beneficial than accepting one. Thus, the latter route is preferred to neutralize radicals. In reality, that is what we see through the RAF values being the lowest. Since RAF is known to dominate in the lipid phase, it is presumed that betalains can react much more strongly in the lipid milieu. Nevertheless, one should bear in mind that aprotic solvent does not allow betalains’ dissociation and hence has an impact on electronic structure that in consequence affects the reactivity, as could be noted from DAM analysis. It is necessary to identify these radical adducts and test their stability, which would confirm the present findings. Overall, even though the Gibbs values of remaining mechanisms are positive, they still have been found to be smaller than for the other common phytochemical antioxidants. This is especially important when dealing with such hard-to-flush species as superoxide and nitric oxide.

Finally, one should keep in mind that the key drawback of the study is a lack or low number of the experimental data, which forced us to operate entirely on the calculations results. Despite the fact that we used validated level of theory, the accuracy of which has been tested [15,56], to estimate the reported properties, these are still quantum chemical methods that may be burdened with an error. Furthermore, although we were unable to fully confirm or reject our conclusions, we did open up a new space for future research on betalains antiradical and antioxidative activity.

## 4. Materials and Methods

The conformers of the selected compounds were generated using semi-empirical method implemented in Gabedit [57]. Afterwards, the lowest energetic representatives were chosen for geometry optimization in Gaussian09 [58] using the M06–2X [56] hybrid functional in Pople’s 6–311++G** basis set [59], identified by the absence of imaginary frequencies. SMD solvent implementation was used to reproduce the water. [60]

Deprotonation routes have been studied for 1 M standard state solutions. Gibbs free energies of deprotonation have been calculated for each compound, at each stage, for each carboxyl and hydroxyl group within it. The lowest energetic pathway was taken into account as symbolic for the compound under investigation and used to calculate p*K*_a_ values as below.

The parameters fitting method (Equation (1)) is the most prominent of the possible methods to set p*K*_a_ values. [61] In this linear regression model, the terms (*m*, *C*_0_) are taken directly from the reference compounds for which p*K*_a_ was experimentally measured. Therefore, it is only appropriate to know the difference in Gibbs free energy between the conjugated base and the corresponding acid (*ΔG*) calculated at the same level of theory. Herein, *m* = 0.318 and *C*_0_ = –82.349 for OH groups, while *m* = 0.358 and *C*_0_ = −95.12 for COOH groups.

(1) $${pK}_{a}=m\Delta G+C_{0}.$$

The Donator–Acceptor Map suggested by Martinez et al. [49] was plotted to compare the overall behavior in the electron transfer-based mechanisms. Vertical ionization potential was calculated as the energy difference between N–1 and N, while vertical electron affinity was calculated as the energy difference between N and N+1 versions of the same electron system (*X*). Afterwards, electrodonating (*ω^–^*) and electroaccepting (*ω^+^*) powers were then estimated (Equations (2) and (3)).

(2) $$\omega_{X}^{-}=\frac{{(3vIP_{X}+vEA_{X})}^{2}}{16(vIP_{X}-vEA_{X})}.$$

(3) $$\omega_{X}^{+}=\frac{{\left(vIP_{X}+3vEA_{X}\right)}^{2}}{16(vIP_{X}-vEA_{X})}$$

From there, the indices of electron donation (*Rd*, Equation (4)) and electron acceptance (*Ra*, Equation (5)) were determined using as the reference electron donating power of sodium $\omega_{Na}^{-}$ and electron accepting power of fluorine $\omega_{F}^{+}$, calculated from the experimental values of their IP and EA, relatively.

(4) $$Rd=\frac{\omega_{X}^{-}}{\omega_{Na}^{-}}.$$

(5) $$Ra=\frac{\omega_{X}^{+}}{\omega_{F}^{+}}.$$

Antiradical potency of betalains has been, in the manner of their reactivity their reactivity indices, explained more generally in the main text. In addition, Gibbs free energies were evaluated for reactions they could have undergone with selected radicals. In the case of radical adduct formation, the preferred attack site was estimated by calculating the condensed Fukui function at given atom (*A*) (Equation (6)):

(6) $$f_{X}^{•}=\frac{q_{A}{(N-1)-q}_{A}\left(N+1\right)}{2}$$

## 5. Conclusions

The antiradical activity of common beetroot betalains has been elucidated. Prior to the main course of the research, the forms favored at physiological pH were identified. For them, an electronic structure analysis was carried out to analyze the deprotonation routes found. A comparative study of the Donator–Acceptor Map was conducted as a computationally cheap and applicable introduction to a more detailed investigation of mechanisms. Six pathways of antiradical activity have been tested for their thermodynamic viability.

Studies on p*K*_a_ have shown that at pH = 7.4 major fractions are represented by the structures with all carboxyl groups dissociated. This means that hydrogen-related channels are not responsible for antiradical activity, unless betanidin and betaxanthin are considered where hydroxyl groups remain untouched. From the outcome of NBO analyses, the deprotonation pattern was associated with strong delocalization of the electron density along conjugated bonds in the core structure.

The electronic structure study outcome suggests that betalains show a modest electron affinity value and not a higher HOMO–LUMO distance value. This means that they are easily oxidized and should willingly accept electrons. Indeed, the Donator–Acceptor Map allocated them to the area favored for antireductant activity.

The estimated Gibbs free energies for hydroxyl, hydroperoxyl, and superoxide radicals, as well as nitric oxide, have established an overall scavenging potency. The RAF mechanism seems to be the most important, but if possible, a two-step pathway—SET–PT or SPLET—would dominate. While the study was performed in the water solvent, the energy values observed for HAT and RAF might be even lower for lipids and drive the reactivity there.

In the end, our findings indicate that betalains have antiradical activity depending on their state. The electron–acceptor channels are more important than electron–donating ones, but either of them has a chance of occurring. If, in reaction with a certain radical, one mechanism represented a high energy value, the other one might exhibit lower values. However, the influence of other solvents and pH is indisputable. In this study, we have confirmed that betalains can act as powerful radical scavengers and thus beneficial inhibitors of oxidative stress development.

## Figures and Tables

**Table 1:** Backbone Structure of Betalains and Studied Common Representatives.

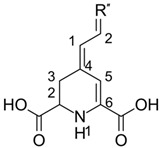	
Compound	R″
Betalamic acid	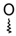
Betanidin ^†^	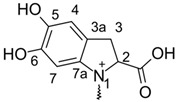
Betaxanthin	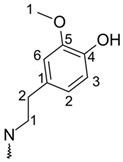
Indicaxanthin	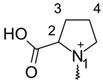
Vulgaxanthin I	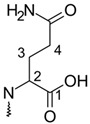
Vulgaxanthin II	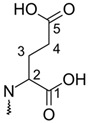

^†^ Betanidin is the only betacyanin; all compounds below it belong to the betaxanthin group.

**Table 2:** p*K*_a_ Values for Each Possible Deprotonation Path. The Numbers in Bold Typeface Indicate the Lowest Value for a Given p*K*_a_.

Compound		p*K*_a1_		p*K*_a2_		p*K*_a3_		p*K*_a4_		p*K*_a5_	
	*H*_2_ *Y*	*→*	*HY^−^*	*→*	*Y* ^2−^						
Betalamic acid	C2	2.96		**3.22**							
	C6	**1.20**		—							

	*H_5_Y^+^*	*→*	*H*_4_ *Y*	→	*H_3_Y^−^*	*→*	*H*_2_ *Y* ^2−^	*→*	*HY* ^4−^	*→*	*Y* ^5−^
Betanidin	C2	1.10		**0.95**		—		—		—	
	C6	**−0.10**		—		—		—		—	
	C2″	0.95		2.08		**2.71**		—		—	
	C5″	6.75		7.00		7.86		**8.14**		—	
	C6″	8.06		8.63		9.41		9.51		**12.00**	

	*H*_3_ *Y*	*→*	*H*_2_ *Y* ^−^	*→*	*HY* ^2−^	*→*	*Y* ^3−^				
Betaxanthin	C2	3.15		**3.69**		—					
	C6	**1.76**		—		—					
	C4″	10.37		10.43		**10.56**					

	*H*_3_ *Y* ^+^	*→*	*H*_2_ *Y*	*→*	*HY^−^*	*→*	*Y* ^2−^				
Indicaxanthin	C2	1.60		2.44		**2.45**					
	C6	0.66		**0.94**		—					
	C2″	**0.59**		—		—					

	*H*_3_ *Y*	*→*	*H*_2_ *Y* ^−^	*→*	*HY* ^2−^	*→*	*Y* ^3−^				
Vulgaxanthin I	C2	2.62		**3.43**		—					
	C6	**1.17**		—		—					
	C1″	4.04		4.19		**4.38**					

	*H*_4_ *Y*	*→*	*H_3_Y^−^*	*→*	*H*_2_ *Y* ^2−^	*→*	*HY^3−^*	*→*	*Y^4−^*		
Vulgaxanthin II	C2	3.21		**3.67**		—		—			
	C6	**0.65**		—		—		—			
	C1″	3.52		3.92		**4.11**		—			
	C5″	— ^†^		5.66		5.50		**6.72**			

^†^ The p*K*_a_ for this carboxyl group cannot be estimated (explanation in the main text).

**Table 3:** Basic Intrinsic Electron Properties.

Compound	vIP [eV]	vEA [eV]	HLG [eV]	ω^–^	ω^+^	Rd	Ra
Betalamic acid	5.39	2.57	2.82	7.79	3.80	2.25	1.12
Betanidin	5.26	3.17	2.09	10.76	6.54	3.11	1.92
Betaxanthin	5.20	1.95	3.25	5.92	2.35	1.71	0.69
Indicaxanthin	5.36	2.89	2.47	9.10	4.98	2.63	1.46
Vulgaxanthin I	5.13	1.99	3.14	6.02	2.46	1.74	0.72
Vulgaxanthin II	5.10	1.95	3.15	5.89	2.37	1.70	0.70

**Table 4:** Gibbs Free Energies of RAF Mechanism. [kcal/mol] ^†^.

$H_{n}X+R^{•}\to \left[H_{n}X-R\right]{}^{•}.$					
Compound		${\mathrm{OH}}^{•}$	${\mathrm{OOH}}^{•}$	$O_{2}^{•-}$	${\mathrm{NO}}^{•}$
Betalamic acid	C1′	–26.4	–1.7	13.8	24.8
	C5	–17.2	6.9	24.8	—
	N1	17.1	41.5	49.3	—

Betanidin	C1′	–26.6	–3.6	10.6	18.8
	C5	–20.2	8.1	23.5	23.1
	C6	–23.5	4.4	—	—

Betaxanthin	C1′	–31.7	–6.4	9.9	18.7
	C5	–21.9	2.4	21.8	24.5
	N1	22.2	—	54.5	—

Indicaxanthin	C1′	–31.0	–5.1	8.5	14.5
	C2′	–27.1	0.1	8.3	27.8
	C5	–16.6	11.5	25.9	22.5

Vulgaxanthin I	C1′	–33.7	–9.7	7.8	13.6
	C5	–22.0	2.0	22.0	17.4
	N1	25.1	—	60.0	—

Vulgaxanthin II	C1′	–33.9	–9.1	8.8	14.4
	C5	–22.7	4.0	25.4	25.3
	N1	20.8	—	54.0	—

^†^ Cases where the system could not reach optimization even after manual displacement into vectors’ direction are indicated by “—”.

**Table 5:** Bond Dissociation Enthalpy and Gibbs Free Energies of PCET Mechanism. [kcal/mol].

$H_{n}X+R^{•}\to H_{n-1}X^{•}+\mathrm{HR}.$						
Compound		BDE	${\mathrm{OH}}^{•}$	${\mathrm{OOH}}^{•}$	$O_{2}^{•-}$ ^†^	${\mathrm{NO}}^{•}$
Betanidin	C5″	72.3	−46.0	−13.1	94.4	23.1
	C6″	77.8	−40.7	−7.9	104.9	28.4

Betaxanthin	C4″	79.9	−38.2	−5.3	126.3	31.0

^†^ The values are presented as the energy of the reaction: $2H_{n}X+O_{2}^{•-}\to 2H_{n-1}X^{•}+H_{2}O_{2}$.

**Table 6:** ET Enthalpy and Gibbs Free Energies of SET Mechanism. [kcal/mol].

$H_{n}X+R^{•}\to H_{n}X^{•+}+R^{-}.$ or $H_{n}X+R^{•}\to H_{n}X^{•-}+R^{+}$						
Compound		ET	${\mathrm{OH}}^{•}$	${\mathrm{OOH}}^{•}$	$O_{2}^{•-}$ ^†^	${\mathrm{NO}}^{•}$
Betalamic acid	•+	102.1	−2.4	19.8	71.9	70.8
	•−	−36.6	166.8	127.1	45.2	88.8

Betanidin	•+	99.2	−4.6	17.6	69.8	68.6
	•−	−50.6	156.3	116.6	34.7	78.3

Betaxanthin	•+	97.7	−6.6	15.6	67.7	66.6
	•−	−22.1	182.9	143.2	61.3	104.9

Indicaxanthin	•+	101.4	−3.0	19.2	71.4	70.2
	•−	−44.0	160.9	121.2	39.3	82.9

Vulgaxanthin I	•+	96.0	−8.5	13.8	65.9	64.8
	•−	−23.2	180.5	140.8	58.9	102.5

Vulgaxanthin II	•+	95.5	−9.2	13.3	65.2	64.0
	•−	−22.1	182.4	142.7	60.7	104.4

^†^ The values are presented as the energy of the reaction: ${2H}_{n}X+O_{2}^{•-}\to 2H_{n-1}X^{•}+H_{2}O_{2}.$

**Table 7:** Gibbs Free Energies of SET–PT Mechanism. [kcal/mol].

$H_{n}X+R^{•}\to H_{n}X^{•+}+R^{-}.$ $H_{n}X^{•+}+R^{-}\to H_{n-1}X^{•}+\mathrm{RH}$											
Compound	aIP		PDE	${\mathrm{OH}}^{•}$		${\mathrm{OOH}}^{•}$		$O_{2}^{•-}$ ^†^		${\mathrm{NO}}^{•}$	
				*ET*	*PT*	*ET*	*PT*	*ET*	*PT*	*ET*	*PT*
Betanidin	99.2	C5″′	11.3	−4.6	−41.4	17.6	−30.8	69.8	−98.0	68.6	−45.5
		C6″	16.7		−36.1		−25.5		−87.6		−40.2

Betaxanthin	97.7	C4″	20.4	−6.6	−31.5	15.6	−20.9	67.7	−78.3	66.6	−35.6

^†^ The values are presented as the energy of the reaction ${2H}_{n}X^{•+}+O_{2}^{2-}\to 2H_{n-1}X^{•}+H_{2}O_{2}.$

**Table 8:** Gibbs Free Energies of SPLET Mechanism. [kcal/mol].

$H_{n}X\to H_{n-1}X^{-}+H^{+}.$ $H_{n-1}X^{-}+R^{•}\to H_{n-1}X^{•}+R^{-}$							
Compound	PA		aIP	${\mathrm{OH}}^{•}$	${\mathrm{OOH}}^{•}$	$O_{2}^{•-}$	${\mathrm{NO}}^{•}$
Betanidin	32.9	C5″	77.7	−26.9	−4.7	47.4	46.3
	36.7	C6″	79.2	−26.0	−3.8	48.4	47.2

Betaxanthin	40.3	C4″	77.8	−26.7	−4.5	47.6	46.5

**Table 9:** Gibbs Free Energies of SPLHAT Mechanism. [kcal/mol].

$H_{n}X\to H_{n-1}X^{-}{+H}^{+}.$ $H_{n-1}X^{-}+R^{•}\to H_{n-2}X^{•-}+\mathrm{HR}$							
Compound	PA		BDE	${\mathrm{OH}}^{•}$	${\mathrm{OOH}}^{•}$	$O_{2}^{•-}{\;}^{†}$	${\mathrm{NO}}^{•}$
Betanidin	32.9	C5″	69.4	−48.9	−16.0	88.6	20.2
	36.7	C6″	65.6	−53.2	−20.3	80.0	15.9

^†^ The values are presented as the energy of the reaction ${2H}_{n-1}X^{-}{+O}_{2}^{•-}\to {2H}_{n-2}X^{•-}{+H}_{2}O_{2}.$

## Data Availability

The data are contained within the article. Additional raw results are available in the supplementary materials.

## Supplementary Materials

The following are available online. Table S1: Gibbs Free Energies of Consecutive Deprotonation Steps, Table S2: Estimated Condensed Fukui Functions (f^•^) for Radical Attack, Table S3: XYZ Coordinates of The Most Stable Betalain Conformer and Its Ion Preferred at Physiological pH.

## Sample Availability

Samples of the compounds are available from the authors.
