# Supplementary material for: Antiradical Activity of Beetroot (Beta vulgaris L.) Betalains
Source: Molecules. 2021 Apr 22;26(9):2439. doi: 10.3390/molecules26092439 (PMC8122748; doi:10.3390/molecules26092439)
Supplement: Supplementary file 1 [file molecules-26-02439-s001.zip › molecules-1151704-supplementary.pdf]

molecules

MDPI

Supplementary Materials

Antiradical Activity of Beetroot (*Beta vulgaris* L.) Betalains

Maciej Spiegel [1,*], Andrzej Gamian [2] and Zbigniew Sroka [1]

[1] Department of Pharmacognosy and Herbal Mediciens, Wroclaw Medical University, Borowska 211A, 50-556 Wroclaw, Poland; maciej.spiegel@student.umed.wroc.pl (M.S.), zbigniew.sroka@umed.wroc.pl (Z.S.)

[2] Ludwik Hirszfeld Institute of Immunology and Experimental Therapy, Polish Academy of Sciences, Rudolfa Weigla 12, 53–114 Wroclaw, Poland; andrzej.gamian@hirszfeld.pl (A.G.)

* Correspondence: maciej.spiegel@student.umed.wroc.pl

Table S1. Gibbs Free Energies of Consecutive Deprotonation Steps. [kcal/mol]

Compound		pK_{a1}	pK_{a2}	pK_{a3}	pK_{a4}	pK_{a5}
Betalamic acid	C2	273.96	274.69			
	C6	269.05	—			
Betanidin	C2	268.77	268.34	—	—	—
	C6	265.42	—	—	—	—
	C2″	268.34	271.51	273.26	—	—
	C5″	280.19	280.96	283.68	284.56	—
	C6″	284.31	286.09	288.55	288,86	
Betaxanthin	C2	274.50	276.01	—		
	C6	270.63	—	—		
	C4″	291.57	291.75	292.18		
Indicaxanthin	C2	267.37	272.50	272.55		
	C6	267.55	268.33	—		
	C2″	270.16	—	—		
Vulgaxanthin I	C2	273.02	275.28	—		
	C6	268.97	—	—		
	C1″	276.99	277.41	277.92		
Vulgaxanthin II	C2	274.65	275.94	—	—	
	C6	267.52	—	—	—	
	C1″	275.53	276.64	277.17	—	
	C5″	—	281.50	281.07	284.48	

Table S2. Estimated Condensed Fukui Functions ($f^{\bullet}$) for Radical Attack.

Compound	N1	C2	C3	C4	C5	C6	C1′	C2′
Betalamic acid	0.22	−0.11	0.10	−0.23	0.29	0.04	0.37	0.01
Betanidin	0.06	−0.02	0.03	−0.05	0.12	0.13	0.19	0.05
Betaxanthin	0.24	0.02	0.27	−0.11	0.03	−0.14	0.33	−0.05
Indicaxanthin	0.12	−0.07	0.11	−0.10	0.21	0.09	0.19	0.19
Vulgaxanthin I	0.20	−0.06	0.14	−0.19	0.28	0.04	0.36	0.37

Vulgaxanthin II	0.23	–0.12	0.11	–0.20	0.29	0.03	0.37	–0.02

Table S3. XYZ Coordinates of The Most Stable Betalain Conformer and Its Ion Preferred at Physiological pH.

Compound	Molecule				Ion			
Betalamic acid	C	-1.0172450000	0.9307000000	1.3517570000	C	0.7055020000	1.2723950000	-1.2922910000
	C	0.1242890000	1.7375290000	0.7240500000	C	-0.6959700000	1.5900450000	-0.7820580000
	N	1.3182500000	0.9190180000	0.6585290000	N	-1.4833820000	0.3725190000	-0.8093360000
	C	1.1475300000	-0.3745580000	0.2235380000	C	-0.9340900000	-0.7526510000	-0.3237510000
	C	-0.0425450000	-1.0189900000	0.1537170000	C	0.4186310000	-0.9028500000	-0.1228140000
	C	-1.2472240000	-0.3644700000	0.6110990000	C	1.3241840000	0.1203030000	-0.5317480000
	C	-2.5072380000	-0.8562290000	0.4993370000	C	2.6891210000	0.1111030000	-0.3621910000
	C	-2.8913270000	-2.1145040000	-0.1226750000	C	3.4692190000	-0.9202140000	0.2588260000
	O	-2.1518670000	-2.8863280000	-0.7203810000	O	3.0576750000	-1.9735090000	0.7583590000
	C	2.3919390000	-1.1408730000	-0.1137450000	C	-1.9303120000	-1.8711150000	0.0176070000
	O	2.4173720000	-2.0265490000	-0.9304520000	O	-1.4575510000	-2.9588230000	0.4096850000
	O	3.5014740000	-0.8045200000	0.5499110000	O	-3.1412030000	-1.5826150000	-0.1261800000
	C	-0.2567190000	2.2468730000	-0.6694030000	C	-0.6647950000	2.2354910000	0.6335880000
	O	0.2899140000	1.9049110000	-1.6869330000	O	-1.4369920000	1.7859040000	1.5101380000
	O	-1.2602290000	3.1293530000	-0.7184230000	O	0.1262650000	3.2018360000	0.7667800000
	H	-1.9317530000	1.5217550000	1.3997960000	H	1.3346720000	2.1586850000	-1.2360870000
	H	-0.7219490000	0.6935800000	2.3785640000	H	0.6381870000	0.9776100000	-2.3458920000
	H	2.1221310000	1.3996220000	0.2637640000	H	-2.4865240000	0.4419140000	-0.6845870000
	H	-0.0609400000	-2.0407700000	-0.1918870000	H	0.7757590000	-1.8230900000	0.3082370000
	H	-3.3252570000	-0.2817240000	0.9208010000	H	3.2556560000	0.9581090000	-0.7340410000
	H	-3.9614310000	-2.3614990000	-0.0305680000	H	4.5544430000	-0.7215570000	0.2782180000
	H	0.3249860000	2.6155660000	1.3410950000	H	-1.1630070000	2.3233160000	-1.4417660000
	H	3.3291090000	-0.1560960000	1.2503240000				
	H	-1.5846940000	3.3686420000	0.1625830000				
Betanidin	C	-1.5680150000	0.6336360000	-1.0040060000	C	-1.6857590000	0.5334390000	-1.0298510000
	C	-2.5067740000	1.2837940000	-0.0005260000	C	-2.6348100000	1.2849720000	-0.1142070000
	N	-3.8692130000	0.7914220000	-0.2210550000	N	-3.9377690000	0.6398770000	-0.1793130000
	C	-3.9872160000	-0.5703910000	-0.4183780000	C	-4.0512560000	-0.6649090000	-0.2569690000
	C	-2.9552880000	-1.3941410000	-0.7085630000	C	-2.9287020000	-1.4962480000	-0.4040480000
	C	-1.6179260000	-0.8634390000	-0.8537290000	C	-1.6917200000	-0.9439270000	-0.7035360000
	C	-0.5626850000	-1.7006510000	-0.9939680000	C	-0.5583880000	-1.7538410000	-0.9460840000
	C	0.8301680000	-1.3508920000	-1.0619530000	C	0.7796390000	-1.4183870000	-0.9057540000
	N	1.4647280000	-0.6021200000	-0.2112460000	N	1.3770020000	-0.4176850000	-0.2400280000
	C	0.9051090000	-0.1037990000	1.0706440000	C	0.8218250000	0.2176350000	0.9779340000
	C	2.1108140000	0.4774110000	1.8547870000	C	2.0108860000	0.9889350000	1.6092980000
	C	3.2548250000	0.3246260000	0.8956590000	C	3.1922790000	0.5665560000	0.7817450000
	C	4.5828660000	0.7000220000	1.0518610000	C	4.5367630000	0.8717680000	0.9361060000
	C	5.4678420000	0.4533190000	0.0138380000	C	5.4471360000	0.3839070000	0.0073430000
	C	5.0341840000	-0.1610410000	-1.1795920000	C	5.0128720000	-0.3940120000	-1.0742060000
	C	3.7131340000	-0.5341610000	-1.3418330000	C	3.6684470000	-0.6975810000	-1.2392280000
	C	2.8542150000	-0.2854160000	-0.2772810000	C	2.7793570000	-0.2189060000	-0.2840400000
	O	5.9164930000	-0.3842710000	-2.1935550000	O	5.9112780000	-0.8577600000	-1.9979200000
	O	6.7886490000	0.7791310000	0.0478500000	O	6.7969040000	0.6255690000	0.0612850000
	C	0.2833480000	-1.2573720000	1.8499050000	C	0.2983450000	-0.8442760000	1.9765830000
	O	-0.6389260000	-0.9081050000	2.7423040000	O	-0.6661810000	-0.4784620000	2.6890190000
	O	0.6352650000	-2.4017310000	1.7403870000	O	0.9105100000	-1.9328070000	2.0456760000
	C	-5.3720880000	-1.1442390000	-0.3686860000	C	-5.4830340000	-1.2264990000	-0.1546280000
	O	-5.5936050000	-2.2991640000	-0.1052140000	O	-5.5981930000	-2.4633420000	-0.2507470000
	O	-6.3783690000	-0.3087580000	-0.6369150000	O	-6.3824320000	-0.3762750000	0.0102500000
	C	-2.5233440000	2.7985910000	-0.1654280000	C	-2.7436530000	2.7856130000	-0.4319150000
	O	-3.0520880000	3.4964150000	0.8438340000	O	-3.8912530000	3.2844650000	-0.4693700000
	O	-2.1124880000	3.3694850000	-1.1420720000	O	-1.6560590000	3.3851010000	-0.5924530000
	H	-0.5686190000	1.0568990000	-0.9362740000	H	-0.6963840000	0.9765250000	-1.0234180000
	H	-1.9328960000	0.8583450000	-2.0129420000	H	-2.0501130000	0.6083640000	-2.0639900000
	H	-2.1853170000	1.0480020000	1.0249640000	H	-2.2757230000	1.2071120000	0.9237590000
	H	2.2892800000	-0.0792520000	2.7774610000	H	2.1341510000	0.7331300000	2.6636910000
	H	1.9260680000	1.5191200000	2.1129760000	H	1.8478760000	2.0656640000	1.5432280000
	H	4.9349480000	1.1799860000	1.9581950000	H	4.8876540000	1.4823640000	1.7616220000
	H	3.3966960000	-0.9911950000	-2.2712470000	H	3.3598110000	-1.2772380000	-2.1002960000
	H	6.7925940000	-0.0662510000	-1.9333880000	H	6.7964940000	-0.5467880000	-1.7633540000
	H	7.0131420000	1.2114780000	0.8824710000	H	7.0033720000	1.2002710000	0.8092390000

	H	0.1663320000	0.6719210000	0.8830750000	H	0.0170490000	0.9068760000	0.7459290000
	H	-0.8373500000	0.0409280000	2.7199100000	H	-4.7762260000	1.2049760000	-0.0689340000
	H	-6.0644810000	0.5563050000	-0.9426300000	H	-3.0617160000	-2.5659710000	-0.3204860000
	H	-3.2810170000	2.9376350000	1.6021260000	H	-0.7506900000	-2.7889880000	-1.2106920000
	H	-4.5466600000	1.1771440000	0.4339750000	H	1.4718500000	-2.0989870000	-1.3941050000
	H	-3.1416390000	-2.4509300000	-0.8489460000				
	H	-0.7530260000	-2.7606810000	-1.1436610000				
	H	1.4448970000	-1.8137110000	-1.8294360000				
	C	-0.0482300000	-2.3381910000	-1.0961370000	C	0.0439580000	-2.3050470000	-1.0590020000
	O	-1.1666120000	-2.6072180000	-0.2534740000	O	-1.0688070000	-2.6118470000	-0.2203750000
	C	-2.1323660000	-1.6429440000	-0.1861680000	C	-2.0537150000	-1.6688080000	-0.1322150000
	C	-3.1759640000	-1.9178200000	0.7080520000	C	-3.0749290000	-1.9702680000	0.7791550000
	C	-4.2118100000	-1.0127080000	0.8558300000	C	-4.1289490000	-1.0902200000	0.9483620000
	C	-4.2124680000	0.1725730000	0.1213990000	C	-4.1714870000	0.0957320000	0.2162680000
	C	-3.1818330000	0.4634270000	-0.7689700000	C	-3.1636350000	0.4132850000	-0.6909580000
	C	-2.1400270000	-0.4613700000	-0.9187830000	C	-2.1011490000	-0.4846780000	-0.8596380000
	C	-3.1868840000	1.7639200000	-1.5441730000	C	-3.2184550000	1.7113630000	-1.4678000000
	C	-3.0587760000	3.0000470000	-0.6459980000	C	-3.1176920000	2.9557300000	-0.5759230000
	N	-1.8640730000	3.1309030000	0.1877790000	N	-1.9211560000	3.1223780000	0.2505700000
	C	-0.8109950000	2.4963010000	-0.1560020000	C	-0.8499910000	2.5266130000	-0.1149900000
	C	0.3957060000	2.5323830000	0.6583870000	C	0.3764240000	2.5810460000	0.6593190000
	C	1.5159280000	1.8330650000	0.3834090000	C	1.4975700000	1.8843870000	0.3488430000
	C	2.7068270000	1.8619840000	1.3074030000	C	2.7148020000	1.9553520000	1.2402510000
	C	3.2667050000	0.4424490000	1.4745480000	C	3.3700480000	0.5762570000	1.3528520000
	N	3.6545800000	-0.0872290000	0.1795960000	N	3.7206890000	0.1193380000	0.0189670000
	C	2.7317910000	0.1168720000	-0.8458640000	C	2.7284350000	0.2027900000	-0.9275740000
	C	1.7111800000	0.9968080000	-0.7936490000	C	1.6585280000	1.0294260000	-0.8128400000
	C	2.9427950000	-0.6531850000	-2.1099200000	C	2.8675800000	-0.7499550000	-2.1136500000
	O	2.0690210000	-0.8489650000	-2.9203680000	O	2.0652610000	-0.6144000000	-3.0666680000
	O	4.1702690000	-1.1383480000	-2.3309300000	O	3.7726470000	-1.6159690000	-2.0234980000
Betaxanthin	C	2.2358130000	-0.4682000000	2.1473770000	C	2.4519710000	-0.4195450000	2.1143010000
	O	1.8657090000	-0.1263000000	3.3870250000	O	2.0724790000	-0.0409550000	3.2526160000
	O	1.7612230000	-1.4497160000	1.6328750000	O	2.1787360000	-1.5183430000	1.5782400000
	O	-3.1764630000	-3.0812950000	1.4358510000	O	-3.0363830000	-3.1359580000	1.5029620000
	H	0.4324730000	-1.4057600000	-0.7966120000	H	0.5045260000	-1.3680710000	-0.7423340000
	H	-0.3615330000	-2.2803800000	-2.1413230000	H	-0.2714200000	-2.2362160000	-2.1030960000
	H	0.6406980000	-3.1697700000	-0.9631310000	H	0.7493070000	-3.1250800000	-0.9410860000
	H	-5.0141750000	-1.2449190000	1.5467450000	H	-4.9122380000	-1.3425620000	1.6539930000
	H	-5.0315920000	0.8730670000	0.2454050000	H	-5.0050560000	0.7760790000	0.3553500000
	H	-1.3357190000	-0.2507340000	-1.6145340000	H	-1.3115750000	-0.2514730000	-1.5654080000
	H	-4.1389840000	1.8670230000	-2.0723750000	H	-4.1796200000	1.7823290000	-1.9851550000
	H	-2.4094230000	1.7473190000	-2.3112840000	H	-2.4508410000	1.7151090000	-2.2447740000
	H	-3.1341860000	3.9010810000	-1.2624790000	H	-3.2253960000	3.8482520000	-1.2004080000
	H	-3.9164340000	3.0314430000	0.0299040000	H	-3.9737280000	2.9649470000	0.1028910000
	H	-0.7748500000	1.8883010000	-1.0587410000	H	-0.8173960000	1.9319530000	-1.0269940000
	H	0.3574700000	3.1262430000	1.5670710000	H	0.3638440000	3.1785000000	1.5664850000
	H	2.4471680000	2.2994520000	2.2713410000	H	2.4528530000	2.3322330000	2.2277110000
	H	3.5009310000	2.4712310000	0.8664570000	H	3.4415800000	2.6437280000	0.7957700000
	H	3.9533340000	-1.0573150000	0.2503590000	H	4.2070940000	-0.7689150000	-0.0193670000
	H	1.0736470000	1.0855210000	-1.6630360000	H	0.9231510000	1.0109910000	-1.6045520000
	H	-2.3774740000	-3.5816990000	1.2193610000	H	-2.2216720000	-3.6092280000	1.2837880000
	H	4.1426250000	0.4702870000	2.1264830000	H	4.2825190000	0.6644370000	1.9456380000
	H	4.8102090000	-0.8340080000	-1.6697770000				
	H	2.3327470000	0.6601780000	3.7062680000				
	C	-4.7364540000	-0.7071900000	-0.5120730000	C	4.6861500000	-0.6552970000	0.6146980000
	C	-3.6034630000	-0.7673150000	-1.5420070000	C	3.5159420000	-0.6026410000	1.6052700000
	C	-2.3752920000	-0.3800840000	-0.7014570000	C	2.3228460000	-0.2718590000	0.6941700000
	N	-2.6295960000	-1.1422650000	0.5314530000	N	2.6063870000	-1.1324560000	-0.4594100000
	C	-1.7890950000	-1.9197370000	1.1389890000	C	1.7880650000	-2.0230610000	-0.9783780000
Indicaxanthin	C	-0.3952990000	-2.1275910000	0.8552160000	C	0.4068310000	-2.1997130000	-0.8022030000
	C	0.5918150000	-1.2312510000	0.6225050000	C	-0.5948670000	-1.2745130000	-0.5175740000
	C	0.5090580000	0.2400260000	0.9066100000	C	-0.4703920000	0.1863790000	-0.8844270000
	C	1.2578370000	1.0554080000	-0.1533910000	C	-1.2922620000	1.0704250000	0.0378010000
	N	2.6298160000	0.5735750000	-0.2702910000	N	-2.6436260000	0.5409410000	0.1291240000
	C	2.8352950000	-0.7918740000	-0.1731670000	C	-2.8752080000	-0.7617310000	0.1276740000

	C	1.9104200000	-1.6836820000	0.2390720000	C	-1.8710540000	-1.6931290000	-0.1038870000
	C	4.2181280000	-1.2806370000	-0.4902970000	C	-4.3251070000	-1.1850390000	0.4206820000
	O	4.4453440000	-2.4014950000	-0.8692090000	O	-4.5394590000	-2.4106740000	0.5147070000
	O	5.2135420000	-0.4067950000	-0.3252990000	O	-5.1514620000	-0.2540670000	0.5376370000
	C	1.2296090000	2.5122790000	0.2885410000	C	-1.3327540000	2.5494840000	-0.3931140000
	O	2.3204820000	2.9867210000	0.8856980000	O	-2.3956810000	3.1731760000	-0.1599310000
	O	0.2506450000	3.2019220000	0.1485780000	O	-0.2911140000	3.0140740000	-0.9063250000
	C	-4.1067760000	-1.2505130000	0.7744510000	C	4.0710690000	-1.2392060000	-0.6667300000
	C	-2.3596150000	1.1420350000	-0.5410200000	C	2.3392920000	1.2407930000	0.3755100000
	O	-2.6789970000	1.7060930000	0.6234040000	O	2.7789260000	1.6138270000	-0.7355120000
	O	-2.1012660000	1.8315400000	-1.4932400000	O	1.9380440000	1.9768170000	1.3081710000
	H	-5.5984260000	-1.3011720000	-0.8079390000	H	5.5096690000	-1.2694180000	0.9754460000
	H	-5.0601270000	0.3253990000	-0.3689970000	H	5.0639420000	0.3506040000	0.4272040000
	H	-3.7404860000	-0.0935720000	-2.3850030000	H	3.6462990000	0.1410160000	2.3900890000
	H	-3.4567470000	-1.7833570000	-1.9102870000	H	3.3522410000	-1.5790170000	2.0656910000
	H	-0.1278590000	-3.1810050000	0.8909180000	H	0.0926130000	-3.2315680000	-0.9287300000
	H	1.0058980000	0.3916300000	1.8734210000	H	-0.8679120000	0.2669710000	-1.9055810000
	H	-0.5055880000	0.6060300000	1.0204340000	H	0.5552350000	0.5331270000	-0.9353280000
	H	3.1307940000	1.0039270000	-1.0461950000	H	-3.4087220000	1.1807300000	0.3153520000
	H	2.1646540000	-2.7347120000	0.2832550000	H	-2.0986120000	-2.7436880000	0.0100970000
	H	-4.3635700000	-0.6740620000	1.6629830000	H	4.3566430000	-0.6823730000	-1.5587930000
	H	-4.3283670000	-2.3020200000	0.9475240000	H	4.3165200000	-2.2915320000	-0.8115240000
	H	0.7306740000	0.9950330000	-1.1119810000	H	-0.8443410000	1.0524260000	1.0438060000
	H	-1.4238460000	-0.6786400000	-1.1428240000	H	1.3610120000	-0.5444220000	1.1298760000
	H	-2.2444650000	-2.5852750000	1.8703650000	H	2.3007450000	-2.7928220000	-1.5525340000
	H	4.9150760000	0.4169510000	0.0916770000				
	H	3.0062230000	2.2968480000	0.9275570000				
	H	-2.8204070000	1.0756940000	1.3456700000				
	C	-2.9242970000	-1.5888030000	0.0007000000	C	-2.9059900000	-1.6403610000	-0.0145690000
	C	-3.6944720000	-0.3498330000	-0.4734200000	C	-3.6980410000	-0.3991230000	-0.4167050000
	N	-3.2509250000	0.8199910000	0.2693100000	N	-3.2251050000	0.7298440000	0.3648920000
	C	-1.8742400000	0.9748280000	0.3388140000	C	-1.8768990000	0.9337830000	0.3976340000
	C	-0.9854710000	-0.0214760000	0.1390160000	C	-0.9770290000	-0.0516450000	0.1328620000
	C	-1.4368050000	-1.3718410000	-0.1501190000	C	-1.4155060000	-1.4025000000	-0.1558220000
	C	-0.6311400000	-2.3949790000	-0.5036580000	C	-0.6053220000	-2.4301750000	-0.5155420000
	C	0.8266840000	-2.3537150000	-0.6410170000	C	0.8487200000	-2.3886180000	-0.6551960000
	N	1.5869400000	-1.6262070000	0.0777780000	N	1.6135310000	-1.6056080000	-0.0002510000
	C	2.9924090000	-1.6270380000	-0.2715710000	C	3.0192120000	-1.5964730000	-0.3545240000
	C	3.3598140000	-0.2925090000	-0.9522500000	C	3.3538950000	-0.2276120000	-0.9719930000
	C	3.1717180000	0.9215140000	-0.0384330000	C	3.2040800000	0.9201990000	0.0348280000
	C	3.4450250000	2.1907110000	-0.8106440000	C	3.3241600000	2.2471610000	-0.6720170000
	O	4.5375440000	2.3816110000	-1.3587850000	O	4.3592950000	2.5598510000	-1.2782790000
	N	2.4432450000	3.0756030000	-0.8801110000	N	2.2507520000	3.0453530000	-0.6335600000
	C	3.8445620000	-1.8146750000	0.9734280000	C	3.9481620000	-1.8511260000	0.8508710000
	O	3.4409790000	-1.7732930000	2.1089060000	O	3.4640400000	-1.9103350000	2.0054300000
	O	5.1530970000	-2.0195220000	0.7545320000	O	5.1703090000	-1.9741440000	0.5678520000
	C	-1.3474820000	2.3112560000	0.7551990000	C	-1.4265440000	2.3509100000	0.7448350000
Vulgaxanthin I	O	-0.2209830000	2.6814770000	0.5194480000	O	-0.1959170000	2.5505020000	0.8987000000
	O	-2.1752860000	3.1182700000	1.4245940000	O	-2.3309480000	3.2118000000	0.8442240000
	C	-5.1881030000	-0.5295780000	-0.2821690000	C	-5.2169250000	-0.5843830000	-0.2893310000
	O	-5.8522050000	0.0901540000	0.5096330000	O	-5.8779130000	0.3320570000	0.2551480000
	O	-5.7728710000	-1.4445260000	-1.0627950000	O	-5.6904100000	-1.6381870000	-0.7840970000
	H	-3.2412620000	-2.4725920000	-0.5536280000	H	-3.2183940000	-2.4952870000	-0.6127630000
	H	-3.1507400000	-1.7501530000	1.0607170000	H	-3.1245830000	-1.8656310000	1.0370500000
	H	-3.7539900000	1.6581440000	-0.0108400000	H	-3.7975550000	1.5643290000	0.3570450000
	H	0.0657810000	0.1933020000	0.2446730000	H	0.0738420000	0.1833570000	0.1686000000
	H	-1.0930800000	-3.3388570000	-0.7781150000	H	-1.0708720000	-3.3704260000	-0.7968820000
	H	1.2432510000	-3.0154960000	-1.4103560000	H	1.2705280000	-3.0983010000	-1.3807060000
	H	3.2471340000	-2.4410210000	-0.9638340000	H	3.2513280000	-2.3698680000	-1.0991870000
	H	2.7183050000	-0.2017220000	-1.8327270000	H	2.6772120000	-0.0665840000	-1.8170350000
	H	4.3935910000	-0.3332540000	-1.3027020000	H	4.3735240000	-0.2469890000	-1.3609150000
	H	2.1665990000	0.9413510000	0.3847170000	H	2.2471860000	0.8524760000	0.5545900000
	H	3.8897520000	0.8884870000	0.7877210000	H	4.0043980000	0.8694060000	0.7799490000
	H	1.5550000000	2.9041980000	-0.4251900000	H	1.4090120000	2.7936720000	-0.1198130000
	H	2.5842860000	3.9422180000	-1.3818760000	H	2.2926390000	3.9385450000	-1.1060870000
	H	-3.5154030000	-0.2154530000	-1.5497970000	H	-3.5059890000	-0.2084040000	-1.4852290000
	H	5.3593060000	-2.0737030000	-0.1905210000				

	H	-3.0044080000	2.6806390000	1.6708250000				
	H	-5.1503530000	-1.8483550000	-1.6854240000				
	C	-3.1016310000	-0.8214210000	-1.5886770000	C	-3.1065340000	-0.9048210000	-1.5814080000
	C	-3.9319400000	-0.1223740000	-0.5023210000	C	-3.9883860000	-0.1787330000	-0.5644550000
	N	-3.3968390000	1.2012300000	-0.2743750000	N	-3.4659030000	1.1623110000	-0.3733250000
	C	-2.0274880000	1.2848030000	-0.1184480000	C	-2.1173980000	1.2739150000	-0.1537260000
	C	-1.1615340000	0.3301910000	-0.5277630000	C	-1.2277180000	0.2989480000	-0.4783090000
	C	-1.6435630000	-0.8652000000	-1.1975890000	C	-1.6624350000	-0.9173640000	-1.1305840000
	C	-0.8747950000	-1.9221910000	-1.5377320000	C	-0.8715240000	-1.9781170000	-1.4321310000
	C	0.5619420000	-2.0679460000	-1.3068800000	C	0.5612680000	-2.1107300000	-1.1862160000
	N	1.1858350000	-1.5558790000	-0.3187380000	N	1.2206210000	-1.4874570000	-0.2877860000
	C	2.6265260000	-1.7023370000	-0.3368480000	C	2.6621470000	-1.6602700000	-0.3064440000
	C	3.2768510000	-0.3698200000	-0.7660900000	C	3.3209250000	-0.3475940000	-0.7673370000
	C	3.0066390000	0.7621450000	0.2352530000	C	3.0888980000	0.8186280000	0.1974430000
	C	3.4535890000	2.0915680000	-0.3068510000	C	3.7418030000	2.0970550000	-0.3231390000
	O	4.4075200000	2.7131940000	0.0994830000	O	4.9952530000	2.0870360000	-0.4694690000
	O	2.7340010000	2.6071300000	-1.3197740000	O	3.0006740000	3.0813230000	-0.5878780000
	C	3.1351190000	-2.0909460000	1.0410440000	C	3.2454230000	-2.0729250000	1.0600850000
	O	2.4929940000	-2.0295860000	2.0594700000	O	2.5008380000	-2.1279900000	2.0673220000
	O	4.4119400000	-2.4993320000	1.0970080000	O	4.4795970000	-2.3322730000	1.0572020000
	C	-1.5854210000	2.5261900000	0.5911330000	C	-1.6657110000	2.5786870000	0.5014600000
Vulgaxanthin II	O	-0.2980370000	2.8708430000	0.5488880000	O	-0.4344920000	2.7890420000	0.5948860000
	O	-2.3688450000	3.2119760000	1.2060440000	O	-2.5814110000	3.3379740000	0.9055710000
	C	-3.9486420000	-0.9361560000	0.7952300000	C	-4.1129710000	-0.9826970000	0.7582380000
	O	-4.5286840000	-2.1415900000	0.7343300000	O	-4.4583720000	-2.1856250000	0.6304620000
	O	-3.4886160000	-0.5512400000	1.8407090000	O	-3.9063990000	-0.3921360000	1.8437780000
	H	-3.2121570000	-0.2321110000	-2.5033230000	H	-3.1692930000	-0.3633290000	-2.5316600000
	H	-3.4811600000	-1.8229790000	-1.7919010000	H	-3.4731030000	-1.9162830000	-1.7486900000
	H	-3.9123570000	1.7632170000	0.3945730000	H	-4.0116870000	1.7702710000	0.2255860000
	H	1.0925710000	-2.6746660000	-2.0511230000	H	1.0754370000	-2.8159190000	-1.8545880000
	H	2.9556990000	-2.4765430000	-1.0417560000	H	2.9562230000	-2.4476930000	-1.0143530000
	H	2.8654510000	-0.1287290000	-1.7491570000	H	2.9156570000	-0.0979380000	-1.7534990000
	H	4.3541530000	-0.5096920000	-0.8828710000	H	4.3928480000	-0.5190070000	-0.8845240000
	H	3.5424170000	0.5913340000	1.1695050000	H	3.5369120000	0.5825990000	1.1693050000
	H	1.9374230000	0.8275960000	0.4530100000	H	2.0213680000	0.9883760000	0.3447920000
	H	-4.9677560000	-0.0406580000	-0.8380930000	H	-5.0015520000	-0.1035310000	-0.9653610000
	H	-0.1006920000	0.4277600000	-0.3496230000	H	-0.1817360000	0.4509090000	-0.2617920000
	H	-1.3343610000	-2.7244180000	-2.1075820000	H	-1.3163200000	-2.7951370000	-1.9932840000
	H	1.9954660000	2.0310030000	-1.5674510000				
	H	4.8186140000	-2.5544110000	0.2192060000				
	H	0.2218360000	2.2998580000	-0.0345890000				
	H	-4.9102140000	-2.3242220000	-0.1370650000				
